# Lack of flow on time-of-flight MR angiography does not always indicate occlusion

**DOI:** 10.1259/bjrcr.20150187

**Published:** 2015-06-10

**Authors:** Grant Mair

**Affiliations:** Centre for Clinical Brain Sciences, University of Edinburgh, Edinburgh, UK

## Abstract

A patient presented acutely with symptoms of cerebellar ischaemia. While non-contrast CT imaging was normal, MRI demonstrated an apparent occlusion of the left vertebral artery on time-of-flight angiography. However, concurrent contrast-enhanced MR angiography (and subsequent CT angiography) demonstrated normal contrast filling of the left vertebral artery. This article discusses the benefits and limitations of time-of-flight angiography for the investigation of possible stroke and highlights a particular technical limitation which could be misinterpreted as an arterial occlusion.

## Summary

A patient presented acutely with symptoms of cerebellar ischaemia. While a non-contrast CT scan was normal, an MRI demonstrated an apparent occlusion of the left vertebral artery on time-of-flight (TOF) angiography. However, a concurrent contrast-enhanced MR angiography (and subsequent CT angiography) demonstrated normal contrast filling of the left vertebral artery.

This article discusses the benefits and limitations of TOF angiography for the investigation of possible stroke and highlights a particular technical limitation that could be misinterpreted as an arterial occlusion.

## Clinical presentation and differential diagnosis

A 45-year-old male patient presented to the emergency department of his local hospital. He described a sudden onset of dizziness, nausea and clumsiness of his legs that came on while repairing his roof. There was no associated fall or collapse. On examination, he was ataxic but neurological examination was otherwise normal. Soon after arrival at the hospital, his symptoms started resolving. He was a smoker and had hypertension.

The working diagnosis was a transient ischaemic attack (TIA) but the differential included other structural abnormalities within the posterior fossa (*e.g*. haemorrhage, mass lesion or demyelination).

## Investigations/imaging findings

**Figure 1.**
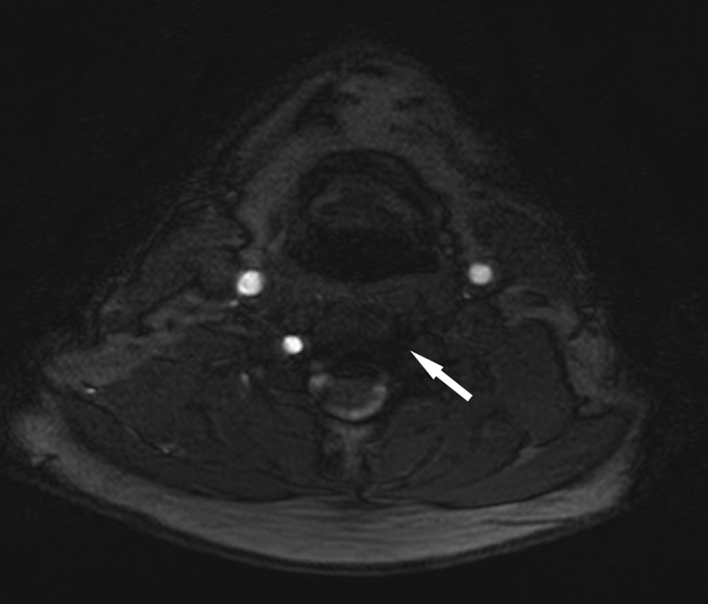
Time-of-flight MR angiography of the arterial neck vessels at the level of the hyoid bone, axial section, shows normal flow signal (white) within both internal carotid arteries (larger anterior vessels) and the right vertebral artery, seen end on. Arrow indicates the expected location of the left vertebral artery but no flow is demonstrated.

**Figure 2.**
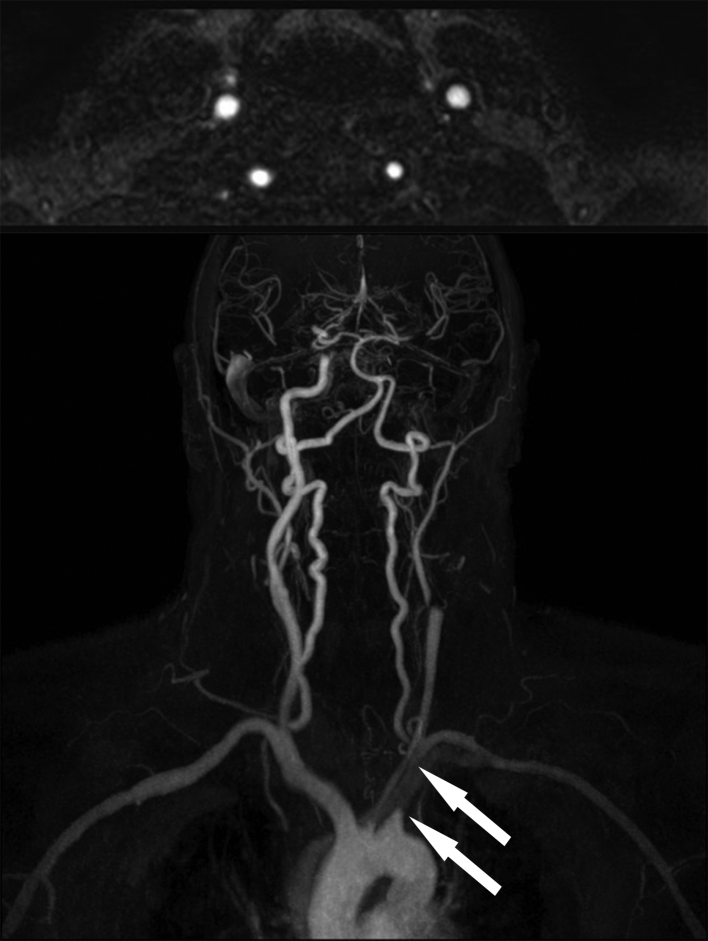
Contrast-enhanced MR angiography of the arterial neck vessels, axial (top image) and coronal (bottom image) sections. Normal flow signal is now demonstrated within the left vertebral artery on the axial section taken at the same location as Figure 1. The coronal section demonstrates a flow gap (arrows) within the proximal left subclavian artery but normal contrast filling of the distal left subclavian artery beyond the origin of the left vertebral artery.

**Figure 3.**
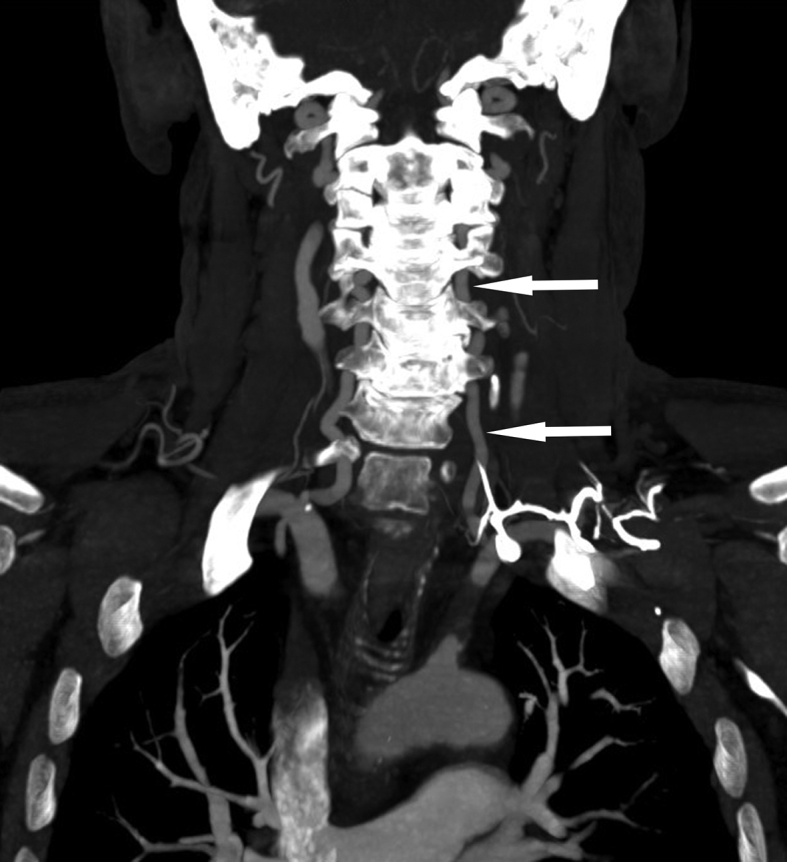
Contrast-enhanced CT angiography of the arterial neck vessels, coronal section. This image better demonstrates the flow gap within the proximal left subclavian artery and again demonstrates normal contrast filling of the left vertebral artery (arrows).

The patient had an immediate non-contrast CT scan of the head performed, which was reported as normal. He was discharged with referral to the local TIA clinic the following day. As part of the TIA work-up, an MRI was performed. No infarct or other posterior fossa abnormality was identified but TOF angiography was abnormal. Figure 1 demonstrates a complete absence of flow within the left vertebral artery (arrow indicates artery location) on TOF angiography. Contrast-enhanced MR angiography was then performed and demonstrated an apparently normal left vertebral artery (Figure 2). However, the contrast-enhanced study also demonstrated occlusion of the proximal left subclavian artery (best seen on the coronal view, arrows indicate the extent of occlusion). Follow-up CT angiography more clearly delineates the subclavian artery occlusion (Figure 3; again the apparently normal contrast opacification of the left vertebral artery within the neck, as indicated by the arrows, is noteworthy).

## Discussion

Proximal occlusion or stenosis of a subclavian artery (most commonly the left)^1^ can result in a subclavian steal syndrome. To maintain blood flow to the arm, the distal subclavian artery “steals” blood from the brain as first described by Reivich and colleagues^2^ in 1961. To explain this phenomenon, consider that with progressive subclavian artery stenosis, blood pressure within the ipsilateral vertebral artery will decrease, and when it drops below the contralateral vertebral arterial blood pressure (which is presumed to be normal), there will be a reversal in the direction of flow within the vertebral artery on the affected side.^3^ In this scenario, blood flow to the arm essentially comes from the contralateral vertebral artery via the basilar artery. This flow reversal can result in reduced perfusion of posterior fossa structures of the brain^4^ and can, rarely, cause transient symptoms of cerebellar and/or brainstem ischaemia (*e.g*. transient ataxia, nystagmus, tinnitus, diplopia, vision loss, vertigo, facial paraesthesia, body hemiparaesthesia or hemiparesis, loss of consciousness); most patients are, however, asymptomatic.^1^ In addition, the patient may experience claudication of the under-perfused affected arm. Ischaemic symptoms are most likely to occur following physical exertion of the affected arm, particularly if the arm is elevated, as in the case provided, but some patients are symptomatic at rest. On examination, there may be a discrepancy in blood pressure measured in the arms, with decreased blood pressure noted on the affected side.^5^ Similarly, the ipsilateral radial and ulnar pulses may be diminished. Most cases of subclavian artery stenosis or occlusion are a consequence of underlying atherosclerotic disease.^6^ Treatment therefore includes tackling risk factors for atherosclerosis in addition to definitive treatment of the arterial stenosis/occlusion such as endovascular angioplasty (± stenting) or surgical vascular bypass grafting.

Doppler angiography of the vertebral arteries should demonstrate arterial flow reversal in patients with a subclavian steal syndrome. Indeed, many cases are incidentally identified this way in asymptomatic patients. However, the altered vertebral artery flow dynamics may need to be stimulated by first exerting the upper limb. It is also noteworthy that bidirectional flow within a vertebral artery does not always indicate a subclavian steal and can be related to hypoplasia or stenosis of the vertebral artery instead.^7^

TOF MR angiography utilizes the movement of blood to produce signal (imaging contrast) without the need for the administration of intravascular contrast material. Signal within the static tissue region of interest (*e.g*. the neck) is saturated or removed through repeated application of radiofrequency excitation pulses with a short repetition time.^8^ Therefore, when the flowing blood enters the volume being imaged, it retains a higher signal (appears brighter) than the surrounding stationary tissue (appears darker), as the blood was not originally within the area of signal saturation; that is, we get signal only from the material that moves into the imaged volume during the scan.^9,10^ To remove the appearance of flowing venous blood (which also moved into the imaged volume during the scan), a further saturation band is placed at the distal end of the imaged volume, that is, just before venous blood enters the volume.^8^ The benefit of this approach is that the signal from venous flow is excluded from the images but so is, as we have seen, the signal from the abnormally reversed arterial flow.

TOF angiography also has a number of other limitations such as:

- It is limited by movement artefact (when imaging the neck vessels, cardiac pulsation usually obscures the aortic arch and the origin of the great vessels).
- It has a reduced ability to detect slow or complex flow or to accurately characterize the extent of an occlusion (TOF techniques cannot differentiate between a standing or slow-moving column of blood and a true occlusion and therefore overestimate both the extent and the length of occlusion).
- Because the source images for TOF are *T*_1_ weighted, the presence of substances that are normally bright on *T*_1_ (*e.g*. blood, fat) can be mistaken for flow signal.
- TOF also has limited spatial resolution and owing to an ever-diminishing signal from moving blood within the imaged volume (the repeated radiofrequency saturation pulses affect even the moving blood), two-dimensional (2D) TOF angiography has a limited field of view [this can be improved with three-dimensional (3D) techniques as discussed below].
- Owing to an inherent need for the flow to be imaged perpendicular to the plane of acquisition, TOF angiography may suffer from signal dropout when imaging tortuous vessels.^8^^–^^10^

Despite these limitations, TOF angiography is often performed first-line for the investigation of cranial and extracranial vascular patency (arterial and venous) using MRI.^10^ As there is no need for intravenous contrast to be administered, TOF angiography is generally simpler and safer than contrast-enhanced studies. In addition, TOF techniques can, if necessary, be repeated within the same visit to the scanner (*i.e*., to provide imaging in different planes or repeat a sequence degraded by movement artefact) without any loss of quality. By imaging multiple thin slices rather than a large volume of tissue,^8^ 3D TOF angiography can improve spatial resolution and reduce some of the artefacts specific to 2D TOF but unfortunately can be associated with further artefacts specific to 3D imaging, for example, the Venetian blind effect.^9^

Contrast-enhanced MR angiography of the neck vessels is often reserved for cases where there is need of greater imaging speed, or spatial resolution, or a greater field of view, or better appreciation of the great vessel origins, or to confirm that an apparent occlusion is real, and, if so, to fully delineate the extent of that occlusion. Intravenous contrast is used to selectively shorten the *T*_1_ of blood relative to other tissues in the imaged volume. Therefore, whether that blood is moving or stationary, or at high or low velocity, and in whichever direction it is flowing relative to the acquisition plane, a good signal is still returned.^8^^–^^10^ No imaging technique is perfect and it is worth noting that contrast-enhanced angiography is limited by a need to accurately synchronize image acquisition with the arrival of the intravenous contrast bolus into the arteries of interest on the first arterial pass of the contrast,^10^ that is, when arterial contrast is maximal and before the venous system is filled with contrast. Such synchronization is significantly more difficult in MRI relative to CT owing to the differences in the speed of acquisition for these modalities; even with ultrafast MRI scanning, it can be challenging. In addition, intravenous contrast will remain in the circulation for a variable amount of time, which precludes immediate repeat angiographic imaging, that is, it needs to be successfully obtained at the first attempt (compare this with TOF angiography that can be repeated as necessary).

Phase-contrast angiography is another non-contrast technique that utilizes the movement of blood to provide imaging contrast. This is achieved by means of two directionally opposite but otherwise identical phase-encoding gradients (that cancel each other out) applied to stationary tissue in the region of interest. Moving blood is affected by only one of the gradients as it enters the field of interest (depending on directionality) and thus generates a signal.^9^ Phase-contrast imaging can be used to identify flow speed as well as direction.^10^ However, phase-contrast imaging is also prone to many artefacts, particularly in the presence of turbulent flow, and, similarly to TOF angiography, tends to overestimate the extent of stenosis. Phase-contrast angiography is not commonly used for assessing the intra- and extracranial arteries.^8,9^

## Learning points

1. Subclavian steal syndrome can occur following proximal occlusion of a subclavian artery; the affected arm “steals” blood by reversing flow in the ipsilateral vertebral artery, which can cause a reduction in perfusion of the brainstem and cerebellum.
2. Signs and symptoms of cerebellar or brainstem ischaemia may occur at rest, or following upper limb exertion, but many patients are asymptomatic.
3. TOF angiography with MRI uses the movement of blood to create signal without the need for intravenous contrast.
4. To isolate arterial flow, TOF angiography suppresses signal from blood moving into the scanned volume from the “venous” end; therefore, a reversal of flow within an artery could be mistaken for an arterial occlusion.
5. Contrast-enhanced angiography with MRI or CT scan is not dependent on flow directionality.

## Consent

Informed consent for the publication of this case report and use of the accompanying images was inferred for the patient as per the institution’s policy. All patients are informed that their images may be used for teaching purposes including publication in peer reviewed journals and are offered the option to opt out.

## References

[1] Labropoulos N, Nandivada P, Bekelis K Prevalence and impact of the subclavian steal syndrome. Ann Surg 2010; 252: 166–70. 20531004 10.1097/SLA.0b013e3181e3375a

[2] Reivich M, Holling HE, Roberts B, Toole JF Reversal of blood flow through the vertebral artery and its effect on cerebral circulation. N Engl J Med 1961; 265: 878–85. 14491362 10.1056/NEJM196111022651804

[3] Potter BJ, Pinto DS Subclavian steal syndrome. Circulation 2014; 129: 2320–3. 24891625 10.1161/CIRCULATIONAHA.113.006653

[4] Taylor CL, Selman WR, Ratcheson RA Steal affecting the central nervous system. Neurosurgery 2002; 50: 679–88; discussion 688–9. 11904017 10.1097/00006123-200204000-00002

[5] Shadman R, Criqui MH, Bundens WP, Fronek A, Denenberg JO, Gamst AC, et al Subclavian artery stenosis: prevalence, risk factors, and association with cardiovascular diseases. J Am Coll Cardiol 2004; 44: 618–23. 15358030 10.1016/j.jacc.2004.04.044

[6] Aboyans V, Kamineni A, Allison MA, McDermott MM, Crouse JR, Ni H, et al The epidemiology of subclavian stenosis and its association with markers of subclinical atherosclerosis: the multi-ethnic study of atherosclerosis (MESA). Atherosclerosis 2010; 211: 266–70. 20138280 10.1016/j.atherosclerosis.2010.01.013 PMC2925848

[7] Chen S-P, Hu Y-P, Fan L-H, Zhu X-L Bidirectional flow in the vertebral artery is not always indicative of the subclavian steal phenomenon. J Ultrasound Med 2013; 32: 1945–50. 24154898 10.7863/ultra.32.11.1945

[8] Raghavan P, Mukherjee S, Gaughen J, Phillips CD Magnetic resonance angiography of the extracranial carotid system. Top Magn Reson Imaging 2008; 19: 241–9. 19512856 10.1097/RMR.0b013e3181a8df26

[9] Vessie EL, Liu DM, Forster B, Kos S, Baxter K, Gagnon J, et al A practical guide to magnetic resonance vascular imaging: techniques and applications. Ann Vasc Surg 2014; 28: 1052–61. 24556317 10.1016/j.avsg.2014.02.001

[10] Ozsarlak O, van Goethem JW, Maes M, Parizel PM MR angiography of the intracranial vessels: technical aspects and clinical applications. Neuroradiology 2004; 46: 955–72. 15580489 10.1007/s00234-004-1297-9

